# High risks of failure observed for A1 trochanteric femoral fractures treated with a DHS compared to the PFNA in a prospective observational cohort study

**DOI:** 10.1007/s00402-021-03824-0

**Published:** 2021-02-26

**Authors:** Max P. L. van der Sijp, Marianne de Groot, Sven A. Meylaerts, Karel J. du Pré, Sander M. Verhage, Inger B. Schipper, Arthur H. P. Niggebrugge

**Affiliations:** 1 Department of Surgery, Haaglanden Medical Centre, P.O. Box 432, 2501 CK The Hague, The Netherlands; 2 Department of Orthopaedics, Haaglanden Medical Centre, P.O. Box 432, 2501 CK The Hague, The Netherlands; 3 Department of Trauma Surgery, Leiden University Medical Centre, P.O. Box 9600, 2300 RC Leiden, The Netherlands

**Keywords:** PFNA, DHS, Stable trochanteric fractures, A1 trochanteric fractures, Implant failure, Dislocation

## Abstract

**Introduction:**

Both the DHS and the PFNA are common and well-studied treatment options for stable trochanteric fractures. The aim of the current study was to compare the implant failure rates of these two implants in 31A1 type trochanteric femoral fractures.

**Materials and methods:**

A single-centre observational cohort study was conducted in the Hip Fracture Unit of a multicentre level 1 trauma teaching hospital between December 2016 and October 2018. Patients with an AO/OTA type 31A1 fracture were included. Pathological fractures, bilateral fractures, high-energy traumas and patients younger than 18 years of age were excluded. Surgery was performed using either a DHS or PFNA. Both were used routinely for stable trochanteric fractures, and allocation was decided by the surgeon performing the operation. The primary outcome of this study was the implant failure rate in the first postoperative year. Secondary outcomes included the reoperation rate, functional recovery, pain and morphine use.

**Results:**

Data were available from 126 patients treated with a DHS (*n* = 32, 25.4%) or PFNA (*n* = 95, 74.6%). Minor differences were observed in the patient characteristics including the prevalence of cognitive impairment (18.8% vs 40.2%; *P* = 0.028), prefracture independence in activities of daily living (87.1% vs 67.4%; *P* = 0.034) and prefracture mobility (independently without aides: 61.3% vs 40.4%; *P* = 0.033). Fractures treated with a DHS showed 25% implant failures, compared to 1.1% for fractures treated with a PFNA (*P* = 0.004). No differences were observed in any of the secondary outcomes.

**Conclusions:**

Significantly more implant failures were observed for the DHS compared the PFNA within 1 year after surgery. Despite the fact that this did not result in differences in revision surgery, we conclude that the PFNA, considering the minimal number of implant-related fractures is a viable implant for A1 type trochanteric fractures.

**Supplementary Information:**

The online version contains supplementary material available at 10.1007/s00402-021-03824-0.

## Introduction

The presence and extent of comminution in trochanteric femoral fractures mainly determine the fracture’s stability and therapeutic challenges. Stable two-part trochanteric fractures are classified as AO/OTA type 31A1 fractures, and unstable trochanteric fractures type 31A2 and 31A3 fractures [16].

The dynamic hip screw (DHS) is long since considered one of the most used treatment options for trochanteric fractures. Recent studies have indicated that intramedullary nails such as the proximal femoral nail antirotation (PFNA) are favourable in terms of providing stability for unstable fractures [15, 16, 18, 29, 32]. For stable trochanteric fractures, however, the DHS is still considered an appropriate implant option [21, 22].

Many studies comparing intramedullary nails and extramedullary implants in trochanteric fractures find only minor differences in implant failure rates. These range between 0 and 6% for the DHS and between 0 and 3% for the PFNA [1, 4, 5, 20, 30]. A systematic review by Parker et al. (2008) comparing intramedullary nails and extramedullary implants for extracapsular hip fractures demonstrated higher rates of surgical complications for intramedullary nails, but included only one study involving the PFNA [17, 18]. Few studies focus specifically on treatment of stable trochanteric fractures, and most of these observed no differences in implant failure and reoperation rates between the PFNA and DHS [5, 20, 30, 31]. Nevertheless, some studies indicated that the PFNA might be favourable in stable trochanteric fractures due to a shorter operation time [5], less blood loss [5, 20], less postoperative pain, faster weight bearing [20] and a better functional recovery in the often frail older hip fracture patient [5, 20, 31].

The observation of a substantial failure rate after DHS treatment of 31A1 fractures in our high-volume hip fracture hospital, prompted the need for further analysis within our Hip Fracture Unit. The aim of the current study was to compare the implant failure rates of the DHS and the PFNA within 1 year after surgery in patients with 31A1 type trochanteric femoral fractures. In addition, other complications, and the potential influence of the implant choice on the long-term independence in activities of daily living were studied.

## Methods

This study was performed and written according to the ‘Strengthening the Reporting of Observational Studies in Epidemiology (STROBE)’ statement guidelines for reporting observational studies [27]. The coded database of the hospital’s Hip Fracture Unit was used for this study. Data were prospectively collected by the treating physicians and nurses. The Hip Fracture Unit is part of a multicentre level 1 trauma teaching hospital, treating approximately 500 proximal femoral fractures annually [23]. All data were handled in agreement with the ‘Code of Conduct for Health Research’ of the Council of the Federation of Medical Scientific Societies. All personal data were handled according to the Dutch Personal Data Protection Act. The methodology of the data collection and of any subsequent observational studies was approved by the institutional Medical Research Ethics Committee (METC Southwest Holland; protocol number 18-029) without the need of individual patient consent due to the observational nature of the study.

### Patients and treatment

All consecutive patients admitted with an AO/OTA type 31A1 trochanteric femur fracture and treated with a DHS or PFNA between December 19, 2016 and October 1, 2018 were included in the study [2]. Patients with AO/OTA type 31A1.1 fractures were not included in this study, as no isolated greater trochanteric fractures were treated with either of the study implants. Diagnostic anterior–posterior (AP) and axial X-rays of the hip and the AP radiograph of the pelvis were made upon admission to the emergency department. Patients with pathological fractures, bilateral fractures, high-energy traumas and patients younger than 18 years of age were excluded. Patient characteristics, including age, sex, health status using the American Society of Anaesthesiologists (ASA) classification were registered during admission. Cognitive impairment was defined as a previously diagnosed form of dementia or a Six Item Cognitive Impairment Test (6CIT) score of ≥ 11 upon admission. The prefracture independence in activities of daily living was rated using the Katz Index of Independence in Activities of Daily Living (Katz ADL), categorized into two groups: independent (0–1) and dependent (3–6) [11]. The prefracture mobility was subdivided in three groups: independent mobility, mobile with a walking aid (walking stick, crutch or stroller) or no independent mobility [26]. Prefracture living situation was grouped as either independent (at home, with or without home care, or at a residency home) or dependent (permanent stay in a nursing home).

The implant devices used were the dynamic hip screw (DHS) with a 2- or 4-hole dynamic compression plate and the short or long proximal femoral nail antirotation (PFNA), both produced by Johnson-Johnson DePuy Synthes. Both the DHS and PFNA were used routinely for fixation of stable trochanteric fractures and allocation of the implant type was decided by the surgeon performing the operation. Surgery was performed in accordance with the national surgical treatment protocol for proximal femoral fractures of the ‘Nederlandse Vereniging voor Heelkunde’ (Dutch Trauma Surgery Society) [25] by an experienced trauma- or orthopaedic surgeon or a resident assisted or supervised by a surgeon. The waiting period from admission to surgery was recorded as well as the operation time (skin-to-skin) and the surgical blood loss (in millilitres as estimated by the surgeon’s team). The Numeric Rating Scale for pain (NRS, 0 meaning ‘no pain’ and 10 the ‘worst pain imaginable’) was rated three times daily during admission for patients without cognitive impairments, and the highest score was recorded, as well as the use of any morphinomimetics at discharge.

The fracture classification of all patients was reassessed by two researchers (MdG, MPL) and updated to the 2018 version of the AO/OTA-classification (Supplemental Digital Content 1) [2]. Disagreements in the classification were resolved through discussion, when necessary with a third researcher (AHP). The quality of the fracture reduction was graded as perfect, acceptable (5°–10° varus/valgus and/or ante- or retroversion, 2–5 mm shortening/displacement ad latum), or poor (> 10 degrees varus/valgus and/or ante- or retroversion, > 5 mm shortening/displacement ad latum) [28, 33] and the tip-apex distance (TAD) was measured using the peroperative fluoroscopy images. A combined TAD (TAD anterior–posterior + TAD axial) of ≤ 20.0 mm was considered an adequate position of the implant type [3].

### Follow-up

All prefracture community dwelling patients (not living permanently in a nursing home) without severe cognitive impairments were requested for routine outpatient check-ups at 6 weeks, 3 months and 1 year after admission. Radiological assessments of the hip were routinely made at 6 weeks and 3 months (and when indicated 1 year after surgery). Patients who were not able to visit the outpatient check-up were contacted by phone. All patients not eligible for the outpatient check-ups were interviewed by phone at the corresponding moments. The information was obtained through a relative, a (professional) caregiver, the general practitioner or nursing home staff if necessary. The present living situation, Katz ADL score, surgical complications, pain (NRS, for patients without severe cognitive impairments) and use of morphinomimetics were recorded.

### Outcome assessments

The primary outcome of this study was the incidence of implant failure in the first postoperative year. Implant failures were assessed and registered by the treating physician, by comparing postoperative radiological examinations to the intraoperative fluoroscopy images at any time within 1 year after surgery. An implant failure was diagnosed when one or more of the following criteria were observed:

- Hyper-dynamization of a dynamic implant (≥ 20 mm [6, 19, 34]).
- Implant cut-outs, through the cortex of the femoral head or neck.
- A malunion or non-union of the fracture leading to patient-reported persistent or progressive pain or loss of function.
- Any other secondary fracture dislocation other than shortening (either varus/valgus, ante- or retroversion) leading to patient-reported persistent or progressive pain or loss of function’.

Radiographic examples of the fractures and complications are provided in Supplemental Digital Content 2.

The secondary outcomes included mechanical failures (breakage of the implant), readmissions (admissions to any hospital due to any cause), reoperations (any surgical intervention on the proximal femur or hip joint at any time after the primary surgery including interventions for deep wound infections and implant removal after fracture healing within 1 year after surgery), revisions (any reoperation performed because of implant or mechanical failures) functional outcomes, pain, morphine use and mortality (death due to any cause within 1 year after surgery).

Recovery of the independence in activities of daily living was defined as a patient-specific Katz ADL score assessed at 6 weeks, 3 months or 1 year after surgery equal to or better than the prefracture (baseline) score.

Blinding was not possible due to the observational nature of the study. All treatment outcomes were documented as part of the routine care by the treating physicians. At the moment of follow-up, the physicians were not aware of the present study.

### Statistical analysis

Patients were grouped according to the implant type (DHS and PFNA). Missing values were not imputed. Means were compared using the independent two-sample *T *test with standard deviations. Medians with interquartile ranges (IQR) and the Mann–Whitney U test were used for data with a non-normal distribution (Kolmogorov–Smirnov test of *P* < 0.05). Categorized characteristics were compared using crosstabs and the Chi-square test if the groups were sufficiently large (expected cell-count < 5) or Fishers-exact test if this condition was not met.

The number of study participants was limited relative to the number of potential confounders. A propensity score (PS) was calculated from all baseline patient characteristics. Multicollinearity of the parameters was first assessed using linear regression collinearity diagnostics (variance inflation factors, VIF > 3.0). The propensity score was used to adjust for potential baseline differences in characteristics between the two cohorts in all multivariate analyses.

Generalized estimating equations (GEE) were used to analyse the recovery of independence in ADL and the use of morphinomimetics at 6 weeks, 3 months and 1 year after surgery. An unstructured marginal model was used for analysing improvement in NRS pain scores and the absolute Katz ADL scores.

A *P *value below 0.05 was considered statistically significant for all final outcomes. All statistical analyses were performed using IBM SPSS Statistics version 25.0 (IBM, Amonk, New York).

### Sample size

This study was performed using a convenience sample from the Hip Fracture Unit of the Haaglanden Medical Centre. All eligible patients were extracted from the total database population. The inclusion period ended on the 1st October 2018, followed by the 1-year follow-up period. This sample size was sufficient to detect a minimal incidence difference of 15% with an estimated failure rate of 1% for the PFNA (alpha, 0.05; beta, 0.2; power, 0.8).

## Results

### Patients and fracture demographics

Between December 21st, 2016 and October 1st, 2018, a total of 126 patients with an AO/OTA type 31A1 proximal femoral fracture were surgically treated with a DHS (*N* = 32, 25.4%, of which 2 (1.6%) with a 4-hole compression plate) or PFNA (*N* = 95, 74.6%). Complete data on all patient characteristics were available for 121 (96.0%) patients.

The mean age of all patients was 81.2 (SD, 12.3) and the majority was female (*N* = 95, 75.4%). No significant differences were observed in the patient characteristics age, sex and ASA classification and prefracture living situation between both treatment groups (Table 1). Patients treated with a DHS had less cognitive impairments (no dementia or abnormal cognitive screening using the 6CIT score: 18.8% vs 40.2%; *P* = 0.028), a better prefracture independence in ADL (Katz ADL ≤ 1: 87.1% vs 67.4%; *P* = 0.034) and a better prefracture mobility (independently without aides: 61.3% vs 40.4%; *P* = 0.033).

**Table 1:** Patient characteristics and treatment aspects per implant type

Characteristic	DHS *N* = 32 (25.4%)	PFNA *N* = 94 (74.6%)	*P* value
*Patient characteristic*			
Mean age (years, SD)	81.3 (8.2)	80.9 (18)	0.97
Sex (f)	23 (71.9)	72 (75.8)	0.59
ASA classification			
I–II	16 (50.0)	36 (38.3)	
III–IV	16 (50.0)	58 (61.7)	0.25
Cognitive impairment	6 (18.8)	37 (40.2)	*0.028*
Activities in daily living			
Independent	27 (87.1)	62 (67.4)	
Dependent	4 (12.9)	30 (32.6)	*0.034*
Mobility			
Independent	19 (61.3)	37 (39.4)	
With walking aid	11 (35.5)	38 (40.4)	
No independent mobility	1 (3.2)	19 (20.2)	*0.033*
Living situation			
Home or residential home	30 (93.8)	77 (81.9)	
Nursing home	2 (6.3)	17 (18.1)	0.15*
*Treatment aspects*			
Time to surgery (h, SD)	21.3 (14.8)	24.8 (23.8)	0.44
Surgeons experience			
Resident	21 (65.6)	61 (64.9)	
Surgeon	11 (34.4)	33 (35.1)	0.94
Operation time (min, IQR)	47 (21)	43 (27)	*0.020*
Surgical blood loss (ml, SD)	100.8 (67.4)	140.2 (108.9)	0.15
TAD > 25 mm	1 (3.3)	3 (3.3)	1.00*
Reduction			
Perfect	6 (22.2)	23 (25.3)	
Acceptable	12 (44.4)	40 (44.0)	
Poor	9 (33.3)	28 (30.8)	0.94

*SD* standard deviation, *IQR* interquartile range, *f *female, *ASA* American Society of Anaesthesiologists, *min* minutes, *ml* millilitre, *Italics* indicate statistical significance (p < 0.05), *TAD* tip-apex distance, mm millimetre, *Fisher’s exact test

No differences were observed in treatment aspects except for the operation time, with a 5-min shorter median operation time for the PFNA (47 min; IQR, 21 vs 43 min; IQR, 27; *P* = 0.020). An inadequate TAD was observed in 13.3% for the DHS and 15.2% for the PFNA (*P* = 1.0). An adequate reduction was achieved in 66.7% of the fractures treated with a DHS and 69.3% for the PFNA (*P* = 0.94). A more detailed overview of fracture reduction and implant positioning by implant type is available in Supplemental Digital Content 3.

### Follow-up

The mean follow-up was 9.8 (SD, 3.9) months. Two patients (both from the DHS group) died before discharge due to non-surgical complications and 20 (15.9%) patients died in total within the 1-year follow-up period. Of all patients, 89 patients (70.6%) were requested to visit the outpatient clinic and 79.5% of the planned visits were attended. 62.7% of all patients attended at least one outpatient check-up. No statistically significant differences were observed in the attendance rates of patients treated with a DHS or a PFNA.

### Outcome parameters

The observed incidence of implant failures was 8 (25%) for the DHS and 1 (1.1%) for the PFNA (*P* < 0.001, Table 2). This difference remained statistically significant (*P* = 0.004) when adjusted for age, sex, ASA classification, premorbid function, living situation and cognitive status.

**Table 2:** Surgical complications per implant type within 1 year after surgery

Surgical complications	DHS *N* = 32	PFNA *N* = 94	*P* value	Adjusted OR (95% CI)	Adjusted *P* value
Implant failure	8 (25.0)	1 (1.1)	< *0.001*	0.04 (0.004–0.35)	*0.004*
Revised	2 (6.3)	1 (1.1)	0.16*	0.25 (0.020–3.06)	0.26
Conservative	6 (18.7)	0 (0.0)			
Mechanical failures	3 (9.4)	0 (0.0)	0.02*	NA	NA
Revised	1 (3.1)	0 (0.0)	0.25*	NA	NA
Conservative	2 (6.3)	0 (0.0)			
Readmissions	9 (28.1)	22 (23.4)	0.64	1.07 (0.40–2.87)	0.89
Reoperations	2 (6.3)	4 (4.3)	0.64*	0.71 (0.11–4.53)	0.72
Mortality	6 (18.8)	14 (14.9)	0.61	0.41 (0.12–1.36)	0.15

^*^Fisher’s exact test. *NA* not available. *Italics* indicate statistical significance (*P* < 0.05)

Mechanical failures were observed after treatment with a DHS in three cases (*N* = 3, 9.4% versus *N* = 0, 0.0% in the PFNA group; *P* = 0.015). All three concerned breakages of cortical screws. Only one patient who had an implant failure and a subsequent mechanical failure underwent a revision operation. Another mechanical failure concerned a single broken cortical screw, and the third patient with a mechanical failure had a revision outside the study’s follow-up period of 1 year.

Upon closer examination of the observed failures, we conclude that the primary reason of failure was a fragile lateral cortex which dislocated or collapsed in 5 of the 8 DHS cases. Two failures in the DHS group had a very long lag screw (> 90 mm measured from the barrel) and collapse of the trochanteric region with an intact lateral cortex, and one was due to a non-union with no identified underlying cause. The failed PFNA concerned a valgus reduction with collapse of the medial trochanteric region and hyper-dynamization of the blade.

No differences between the DHS and PFNA groups were found in the incidence of revisions (6.3% vs 1.1%; adjusted *P* = 0.28), readmissions (28.1% vs 23.4%; adjusted *P* = 0.89), reoperations (6.3% vs 4.3%; adjusted *P* = 0.72) and mortality (18.8% vs 14.9%; adjusted *P* = 0.15).

**Fig. 1:**
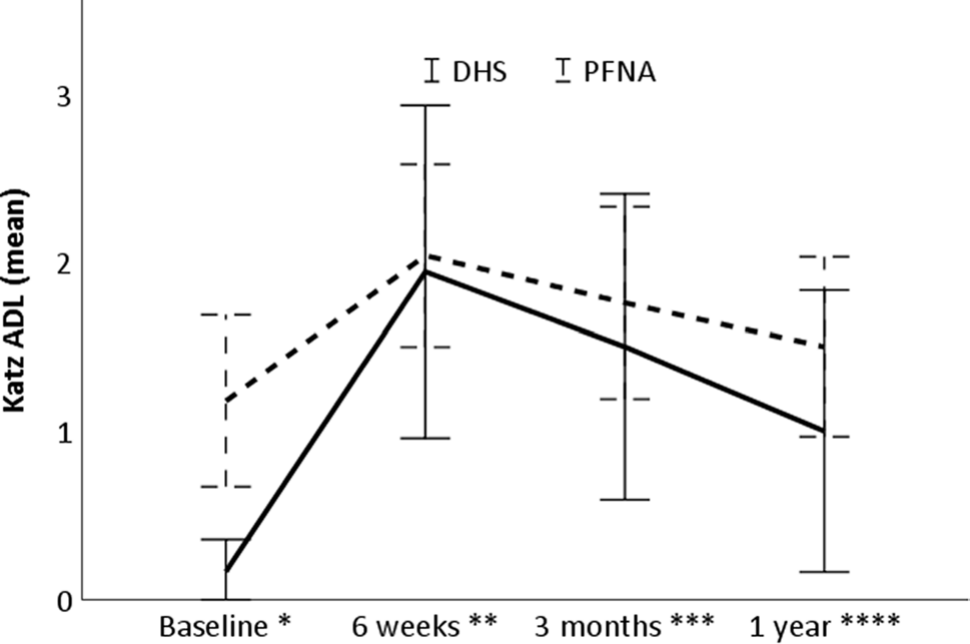
Mean Katz ADL scores for patients treated with a DHS and PFNA. ADL activities of daily living, Adj adjusted. Error bars indicate the unadjusted 95% confidence intervals. Baseline represents the prefracture Katz ADL. *N* = 123. *P = 0.029, adj *P* = 0.90; **P = 0.90, adj *P* = 0.25; ***P = 0.76, adj *P* = 0.33, ****P = 0.19, adj *P* = 0.86; ADJUSTED repeated measure coefficient estimate: 0.27; 95% CI − 0.52 to 1.06; *P* = 0.50

**Fig. 2:**
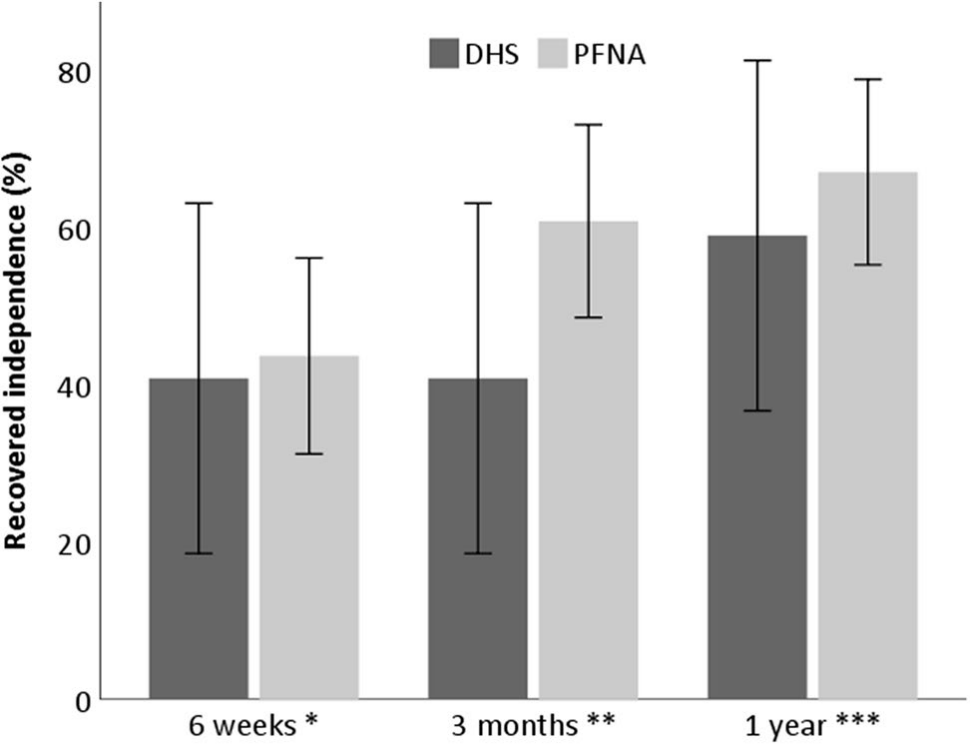
Percentage recovered to their individual prefracture level of ADL for patients treated with a DHS and PFNA. Adj adjusted. Error bars indicate the unadjusted 95% confidence intervals. *N* = 105. *P = 0.68, adj *P* = 0.67; **P = 0.12, adj *P* = 0.087; ***P = 0.64, adj *P* = 0.40; adjusted repeated measure Exp(B), 0.80; 95% CI − 0.36 to 1.80; *P* = 0.60

**Fig. 3:**
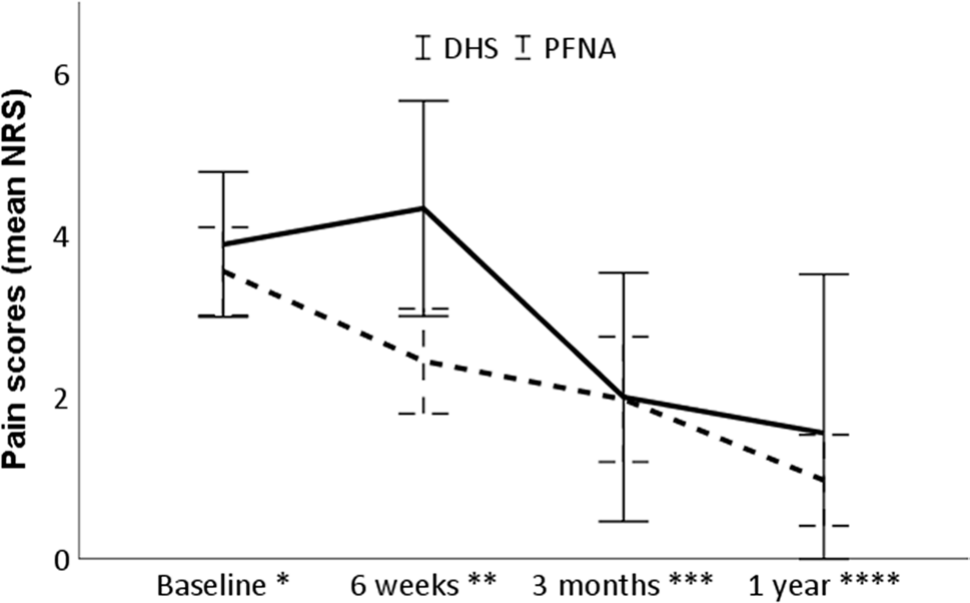
Pain scores of patients treated with a DHS and PFNA. Adj adjusted. Error bars indicate the unadjusted 95% confidence intervals. Baseline represents the maximum postoperative in-hospital pain score. *N* = 105. *P = 0.70, adj *P* = 0.46; ** P = 0.16, adj *P* = 0.12; ***P = 0.74, adj *P* = 0.78, ****P = 0.51, adj *P* = 0.53; adjusted repeated measure estimate, 0.25; 95% CI − 0.89 to 1.39; *P* = 0.66

**Fig. 4:**
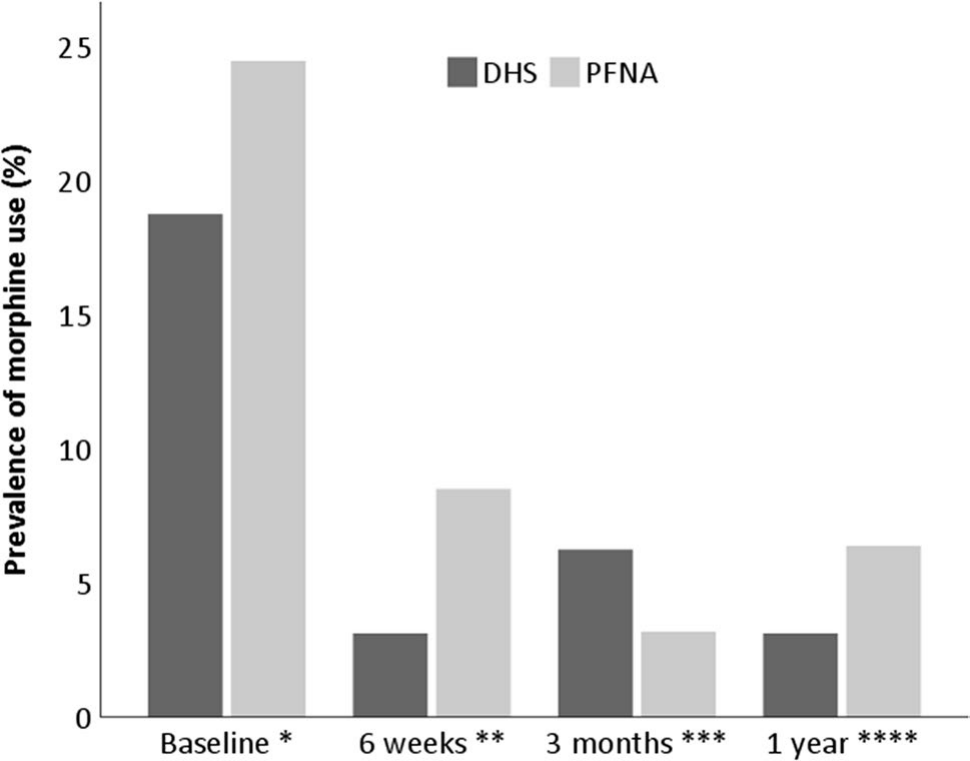
Use of morphine in patients treated with a DHS and PFNA. *N* = 126. Adj adjusted. *P = 0.51, adj *P* = 0.85; **P = 0.45, adj *P* = 0.45; ***P = 0.60, adj *P* = 0.27, ****P = 0.68, adj *P* = 0.82; adjusted repeated measure Exp(B), 0.98; 95% CI 0.46–2.10; *P* = 0.95

Improvement in independency over time was observed for both groups, without significant differences in the scores at 6 weeks, 3 months and 1 year after surgery (adj repeated measure Exp(B), 0.27; 95% CI − 0.52 to 1.06; *P* = 0.50; *N* = 123, Fig. 1, Appendix Table 3). Of the assessed patients 41.1%, 55.6% and 66.7% successfully recovered to their prefracture level of independence in ADL at 6 weeks, 3 months and 1 year, respectively, without significant differences between the DHS and PFNA (adj repeated measure Exp(B), 0.80; 95% CI − 0.36 to 1.80; *P* = 0.60; *N* = 105, Fig. 2). No statistically significant differences were observed in the postoperative pain scores (estimate, 0.25; 95% CI − 0.89 to 1.39; *P* = 0.66; *N* = 105, Fig. 3, Appendix Table 4) or the prevalence of morphine use up to 1 year after surgery either (Exp(B), 0.98; 95% CI 0.46–2.10; *P* = 0.95; *N* = 126, Fig. 4).

## Discussion

Significantly more implant failures were observed for A1 stable trochanteric fractures treated with a DHS compared to those treated with a PFNA within 1 year after surgery, also after adjusting for potential confounders.

While a 25% failure rate was observed for the DHS, the same group of experienced surgeons achieved a failure rate of only 1.1% for the PFNA in similar A1 fracture types. No significant role of the surgical reduction on the implant failure rate could be determined by this study. Although a lower rate of unfavourable reductions was observed amongst patients treated with a DHS, an unfavourable reduction could in theory be more prone to implant failures in fractures treated with a DHS versus a PFNA. In this sense, the PFNA would be a more forgiving implant than the DHS, and could be a partial explanation for the major difference in the failure rate.

Routine radiographic examinations may in some cases underestimate the instability of an A1 trochanteric fracture, and preoperatively falsely classify an A2 fracture as an A1 fracture. A study by Van Embden et al. [24], however, showed that even additional CT scans of trochanteric fractures did not lead to an improved agreement in the surgeons’ fracture classification and implant choice. Alternatively, drilling and positioning an implant or postoperative loading of the hip in a fracture with a non-diagnosed compromised lateral wall might also affect the fractures’ characteristics and increase instability.

When considering use of a DHS for A1 fractures, apart from meticulously inspecting the integrity of the lateral wall on high-quality radiographic examinations, special attention should be paid to bone quality, strength of the lateral cortex, achieving an adequate intraoperative reduction and careful positioning of the drill hole in the lateral cortex [9].

Most previous studies reported failure rates for sliding hip screw devices lower than those observed in our study, ranging from 0 to 6%. Only Yu et al. presented failure rates in the order of magnitude observed in our cohort [1, 4, 5, 20, 31]. While Adams et al. (2001) reported only failures which were reoperated, some studies stated less clearly whether non-reoperated failures were also included as implant failures [4, 20]. Only Cho et al. (2016) and Yu et al. (2016) clearly included these non-reoperated failures also [5, 31]. Reoperation rates were not reported by all studies [4, 5, 20]. The reported rates from studies that did, were similar to those found in this study [1, 31]. This could imply that the high observed failure rate compared to these previous studies has resulted from a discrepancy in what was classified as an implant failure. The definitions of the other studies, compared to the one of this study, seems either too vague to be sure [1, 4, 20], or seems to match reasonably [5, 31].

Other potential reasons for the significant discrepancy in the failure rates, such as differences in the patient populations, cannot be ruled out. Considerable fewer patients with severe cognitive impairments were observed in the study by Cho et al. [5], which had a minimum follow-up of 1 year and might cause a survival bias in favour of fitter patients. For randomised controlled trials, inclusion with informed consent might be challenging for the frailest patients with severe cognitive impairments, which could also have caused a selection bias towards fitter patients in these studies [4, 20]. Frail patients with higher risks of poor bone quality, however, may be more prone to failures. Carulli et al. [4] also included ‘stable and rather stable’ A2 type fractures. The method and length of the follow-up period could be another source of heterogeneity in the failure rate of this study compared to other observational studies [5]. Not all studies used routine radiological follow-ups for all patients [1]. Compared to the routine care of other local hospitals and other observational studies of proximal femoral fracture surgery, the attendance rate of this studies’ routine outpatient clinic for postoperative check-ups is regarded high. Other reasons could include the use of different implant versions, the quality of reduction and subjective aspects of physicians assessing the outcomes. These aspects limit the comparability of outcomes.

Due to the small sample size and the associated statistical limitations, no statistically significant differences were observed for any secondary outcomes, which may have been associated with the failure rates. Reoperations and revision rates did not differ significantly, and we hypothesise that in this patient population, the choice for revision surgery or conservative treatment of the implant failure depends heavily on the functionality and physical condition of the patient. Consequently, for older patients with severe comorbidities, a more reserved approach is frequently accepted.

### Limitations

Limitations inherent to observational studies do apply. Differences in the baseline characteristics cognition, independence and mobility between the two cohorts were observed. This indicates possible confounding by indication, potentially by an association between worse prefracture status and worse bone quality, thus opting for a more stable implant [8, 13]. However, assuming this only further substantiates the notion that the PFNA is less prone to fail compared to the DHS. A propensity score was used to adjust for the baseline differences. Because many patient characteristics relevant for the outcomes of fracture patients were included in the propensity score, an acceptable validity of these outcomes is expected [12].

At 1 year, X-rays were only taken when patients experienced hip pain or disability. This may have caused a potential underestimation in the actual failure rate as described in this study. However, since implant failures are practically always accompanied with pain symptoms, and since pain was in part a condition of failures in our primary outcome, we expect few unidentified failures. In addition, little effect on any of the clinically relevant secondary outcomes is expected from a failure without any pain or disability experienced by the patient.

The study may well be underpowered to observe any clinical consequences of the implant types. Pain scores were only available from patients without severe cognitive impairments and may not reflect outcomes representative for the whole population. No major differences were observed in the implant position or fracture reduction between the two implants, but the sample size is too small for a more in-depth analysis. Whether the difference in implant failure rate can mainly be attributed to a technically more difficult positioning of the implant device, retaining a good reduction during implantation or because one implant device is more forgiving than the other regarding the reduction, could be explored more in-depth by larger studies.

Although intramedullary nails are sometimes generalized, no definitive conclusions can be drawn for any other types besides the PFNA from this study [14, 29]. Using different methods to assess functionality or recovery, for example using the Barthel index instead of the Katz ADL, may yield different results [7, 10].

Additional research using a multicentred trial or a nationwide database, or exploration of the cost differences and clinical outcomes could be used to further substantiate the conclusions of this study.

## Conclusion

Significantly more implant failures were observed for fractures treated with a DHS compared to those treated with a PFNA within 1 year after surgery. Despite the fact that this did not result in differences in revision surgery, we conclude that the PFNA, considering the minimal number of implant-related fractures is a viable implant for A1 type trochanteric fractures.

### Supplementary Information

Below is the link to the electronic supplementary material.

Supplementary file1 (DOCX 459 KB)

Supplementary file2 (DOCX 614 KB)

Supplementary file3 (DOCX 17 KB)

## Appendix

See Tables 3 and 4.

**Table 3:** Mean Katz ADL scores for patients treated with a DHS and PFNA

Time	Implant	Katz ADL, mean (SD)	Mean diff (95% CI)	*P* value	Adj *B* coef (95% CI)	Adj *P* value
Baseline	DHS	0.81 (1.64)	− 0.88 (− 1.66;− 0.09)	*0.029*	− 0.04 (− 0.88 to 0.60)	0.90
	PFNA	1.68 (2.00)				
6 weeks	DHS	1.95 (1.96)	− 0.06 (− 1.00 to 0.88)	0.90	− 0.52 (− 1.41 to 0.36)	0.25
	PFNA	2.02 (1.86)				
3 months	DHS	1.86 (1.95)	− 0.16 (− 1.20 to 0.87)	0.76	− 0.47 (− 1.41 to 0.474)	0.33
	PFNA	1.85 (2.01)				
1 year	DHS	1.20 (1.85)	− 0.63 (− 1.56 to 0.31)	0.19	− 0.08 (− 0.91 to 0.76)	0.86
	PFNA	1.83 (2.03)				

Adjusted repeated measure coefficient estimate: 0.27; 95% C − 0.52 to 1.06; *P* = 0.50. *Italics* indicate statistical significance (*P* < 0.05), *ADL* activities of daily living, *SD* standard deviation, *Diff* difference, *Adj* adjusted, *Coef* coefficient

**Table 4:** Pain scores of patients treated with a DHS and PFNA

Time	Implant	NRS pain, mean (SD)	Mean diff (95% CI)	*P* value	Adj *B* coef (95% CI)	Adj *P* value
Baseline	DHS	3.46 (1.37)	− 0.13 (− 0.82 to 0.55)	0.70	− 0.04 (− 0.88 to 0.60)	0.90
	PFNA	3.60 (1.63)				
6 weeks	DHS	3.32 (1.96)	0.79 (− 0.32 to 1.89)	0.16	− 0.52 (− 1.41 to 0.36)	0.25
	PFNA	2.53 (2.08)				
3 months	DHS	1.71 (2.00)	− 0.21 (− 1.49 to 1.06)	0.74	− 0.47 (− 1.41 to 0.474)	0.33
	PFNA	1.92 (2.35)				
1 year	DHS	1.27 (2.37)	0.39 (− 0.80 to 1.57)	0.51	− 0.08 (− 0.91 to 0.76)	0.86
	PFNA	0.88 (1.56)				

Adjusted repeated measure estimate, 0.25; 95% CI − 0.89 to 1.39; *P* = 0.66. *Italics* indicate statistical significance (*P* < 0.05), *ADL* activities of daily living, *SD* standard deviation, *Coef* coefficient
