# Supplementary material for: High risks of failure observed for A1 trochanteric femoral fractures treated with a DHS compared to the PFNA in a prospective observational cohort study
Source: Arch Orthop Trauma Surg. 2021 Feb 26;142(7):1459–67. doi: 10.1007/s00402-021-03824-0 (PMC9217838; doi:10.1007/s00402-021-03824-0)
Supplement: Supplementary file 1 — Supplementary file1 (DOCX 459 KB) [file 402_2021_3824_MOESM1_ESM.docx]

**Supplemental Digital Content 1.** The 2018 version of the AO/OTA fracture classification for 31A1 fractures.


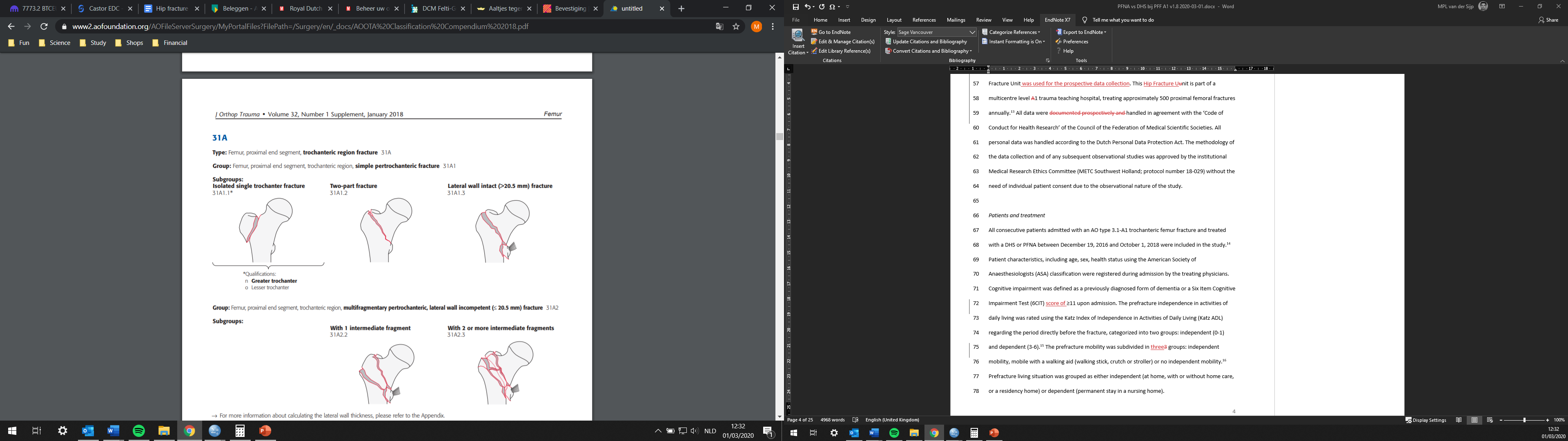


Reference: Fracture and Dislocation Classification Compendium—2018.
