# Supplementary material for: High risks of failure observed for A1 trochanteric femoral fractures treated with a DHS compared to the PFNA in a prospective observational cohort study
Source: Arch Orthop Trauma Surg. 2021 Feb 26;142(7):1459–67. doi: 10.1007/s00402-021-03824-0 (PMC9217838; doi:10.1007/s00402-021-03824-0)

**Supplemental Digital Content 2.** Radiographic examples of fractures, the implants directly after implantation and the complication.


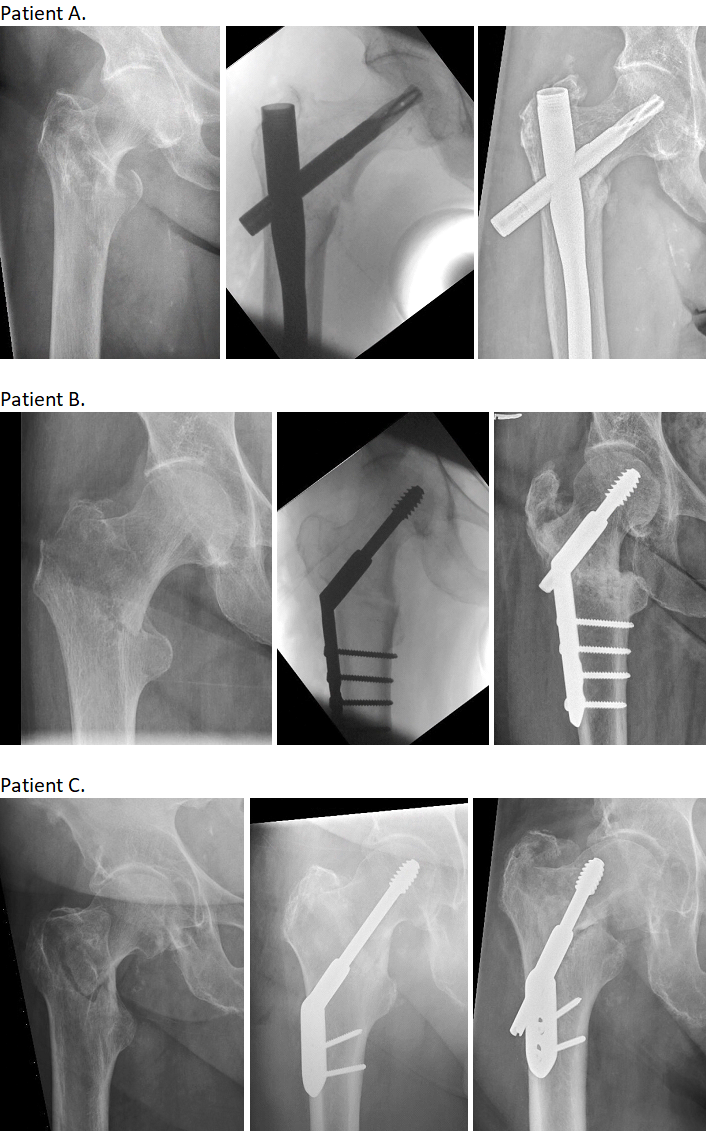

Supplement: Supplementary file 2 — Supplementary file2 (DOCX 614 KB) [file 402_2021_3824_MOESM2_ESM.docx]
