# Supplementary material for: High risks of failure observed for A1 trochanteric femoral fractures treated with a DHS compared to the PFNA in a prospective observational cohort study
Source: Arch Orthop Trauma Surg. 2021 Feb 26;142(7):1459–67. doi: 10.1007/s00402-021-03824-0 (PMC9217838; doi:10.1007/s00402-021-03824-0)
Supplement: Supplementary file 3 — Supplementary file3 (DOCX 17 KB) [file 402_2021_3824_MOESM3_ESM.docx]

**Supplemental Digital Content 3.** Fracture reduction and implant placement details per implant type and the occurrence of implant failure.

| **Implant type** |  |  | **No implant failure** | **Implant failure** | | |
| --- | --- | --- | --- | --- | --- | --- |
| **DHS** | Reduction |  |  |  |  |  |
|  |  | Perfect | 4 (66.7) | 2 (33.3) |  |  |
|  |  | Acceptable | 7 (58.3) | 5 (62.5) |  |  |
|  |  | Poor | 8 (42.1) | 1 (12.5) |  |  |
|  | TAD |  |  |  |  |  |
|  |  | Adequate | 22 (75.9) | 7 (24.1) |  |  |
|  |  | Inadequate | 1 (100.0) | 0 (0.0) |  |  |
| **PFNA** | Reduction |  |  |  |  |  |
|  |  | Perfect | 22 (95.7) | 1 (4.3) |  |  |
|  |  | Acceptable | 40 (100.0) | 0 (0.0) |  |  |
|  |  | Poor | 28 (100.0) | 0 (0.0) |  |  |
|  | TAD |  |  |  |  |  |
|  |  | Adequate | 88 (98.9) | 1 (1.1) |  |  |
|  |  | Inadequate | 3 (100.0) | 0 (0.0) |  |  |

TAD mean tip-apex distance in millimetres, AP anterior-posterior, Ax axial.
